# Prognostic and therapeutic prediction by screening signature combinations from transcriptome–methylome interactions in oral squamous cell carcinoma

**DOI:** 10.1038/s41598-022-15534-7

**Published:** 2022-07-06

**Authors:** Congyu Shi, Shan Liu, Xudong Tian, Cheng Miao, Renyi Wang, Xiangrui Ma, Xiaoyi Wang, Yubin Cao

**Affiliations:** 1 State Key Laboratory of Oral Diseases, National Clinical Research Center for Oral Diseases, Sichuan University, Chengdu, 610041 China; 2 Department of Head and Neck Oncology, West China Hospital of Stomatology, Sichuan University, Chengdu, 610041 China; 3 Department of Oral and Maxillofacial Surgery, West China Hospital of Stomatology, Sichuan University, No. 14, Section 3, South Renmin Road, Chengdu, 610041 China; 4 Department of Oral and Maxillofacial Surgery, Binzhou Medical University Hospital, Binzhou, 256603 China

**Keywords:** Oral cancer, Data integration

## Abstract

DNA methylation pattern in oral squamous cell carcinoma (OSCC) remains poorly described. This study aimed to perform a genome-wide integrated analysis of the transcriptome and methylome and assess the efficacy of their prognostic signature model in patients with OSCC. We analyzed transcriptome and methylome data from 391 OSCC samples and 41 adjacent normal samples. A total of 8074 differentially expressed genes (DEGs) and 10,084 differentially expressed CpGs (DMCpGs) were identified. Then 241 DEGs with DMCpGs were identified. According to the prognostic analysis, the prognostic signature of methylation-related differentially expressed genes (mrDEGPS) was established. mrDEGPS consisted of seven prognostic methylation-related genes, including *ESRRG*, *CCNA1*, *SLC20A1*, *COL6A6*, *FCGBP*, *CDKN2A*, and *ZNF43*. mrDEGPS was a significant stratification factor of survival (*P* < 0.00001) irrespective of the clinical stage. The immune effector components, including B cells, CD4^+^ T cells, and CD8^+^ T cells, were decreased in the tumor environment of patients with high mrDEGPS. Immune checkpoint expressions, including CTLA-4, PD-1, LAG3, LGALS9, HAVCR2, and TIGHT, were comprehensively elevated (*P* < 0.001). The estimated half-maximal inhibitory concentration difference between low- and high-risk patients was inconsistent among chemotherapeutic drugs. In conclusion, the transcriptome–methylome interaction pattern in OSCC is complex. mrDEGPS can predict patient survival and responses to immunotherapy and chemotherapy and facilitate clinical decision-making in patients with OSCC.

## Introduction

Oral squamous cell carcinoma (OSCC) is one of the most common malignancies in the head and neck region, which impairs the quality of life^1,2^. Over the past 30 years, the age-standardized incidence rate was 6.2 and 3.6 per 100,000 for males and females, respectively, and the age-standardized death rate was 3.3 and 1.6 per 100,000 for males and females, respectively^3,4^. However, the 5-year survival rate after surgery or chemoradiotherapy was only 64.4%, according to the 8^th^ edition of the American Joint Committee on Cancer (AJCC), with age of the patient and stage of OSCC as independent prognostic factors^5^. For early stage OSCC, the survival rate did not significantly increase (69.7%), which indicated the difficulty of survival modification in patients with OSCC^6^. With the inclusion of the depth of invasion and extranodal extension, the 8th edition AJCC staging exhibited superior performance in stratifying the survival of patients with OSCC than that by the former edition^7^. However, stratifying the patient survival remained significantly challenging.

Recently, it has become evident that epigenetic mis-programming constitutes a core component of cancer initiation and progression^8^. Currently, DNA methylation remains the main epigenetic marker that can be measured reliably using genome-wide studies in large numbers of samples^9^. DNA methylation occurs almost exclusively in CpG dinucleotides. The CpG dinucleotides tend to cluster in regions called CpG islands (CGI), while most tissue-specific differentially methylated regions appear outside of CGIs^10^. A comprehensive study of the profiles of different healthy individuals and tissue types enables the estimation of variance of each CpG site in the methylome^11^. The integrated analysis of methylome and clinical data made prognostic classification feasible based on methylome analysis in colorectal cancer, hepatocellular carcinoma, and leukemia^12–14^.

Moreover, there may be a link between DNA methylation and the tumor microenvironment^15^. Infiltrating immune cells are important participants in the tumor microenvironment (TME); they are involved in proliferation, signal maintenance, cell death resistance, invasion, metastasis, and angiogenesis^16^. Tumor-associated macrophages support disease progression and resistance to therapy by providing malignant cells with trophic and nutritional support^17^. Altered signals from tumor cells produce a suppressive tumor microenvironment for enrichment of inhibitory cells^18^. DNA methylation may participate in the changes of infiltrating immune cells, reflecting a specific immune response to the cancer cell^19^. Immune TME (TIME) is associated with therapy responsiveness of the immune-checkpoint blockade^20^.

Previous studies have preliminarily depicted the roles of DNA methylation in gene expression, patient prognosis, and immune markers in head and neck cancers^21–23^. However, a comprehensive analysis of transcriptome–methylome interactions and clinical characteristics may be required to mine the potential of methylation orchestration in the management of OSCC. Therefore, this study aimed to perform a genome-wide integrated analysis of the transcriptome and methylome, depict the complex pattern between methylation and gene expression, identify methylation-related differentially expressed genes (mrDEGs), and assess the efficacy of their prognostic signature model in predicting patient survival, TIME alterations, and responses to immunotherapy and chemotherapy in patients with OSCC.

## Results

### Patterns of DMCpGs and differential gene expression (DEG)

**Figure 1:**
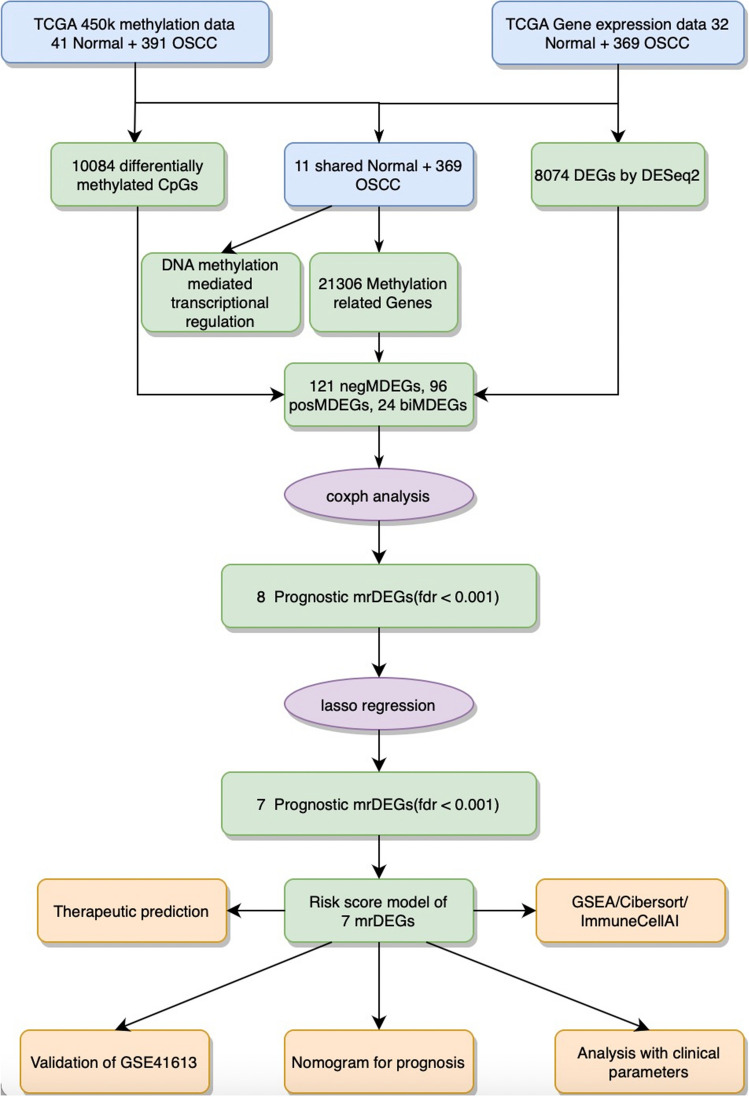
Flow chart of this study. OSCC, oral squamous cell carcinoma; DEGs, differentially expressed genes; DMCpGs, differentially expressed CpGs; negMDEGs, DEGs with positively related DMCpGs; posMDEGs, DEGs with negatively related DMCpGs; biMDEGs, DEGs with bidirectional (both positively and negatively related) DMCpGs; mrDEGs, methylation-related differentially expressed genes.

**Figure 2:**
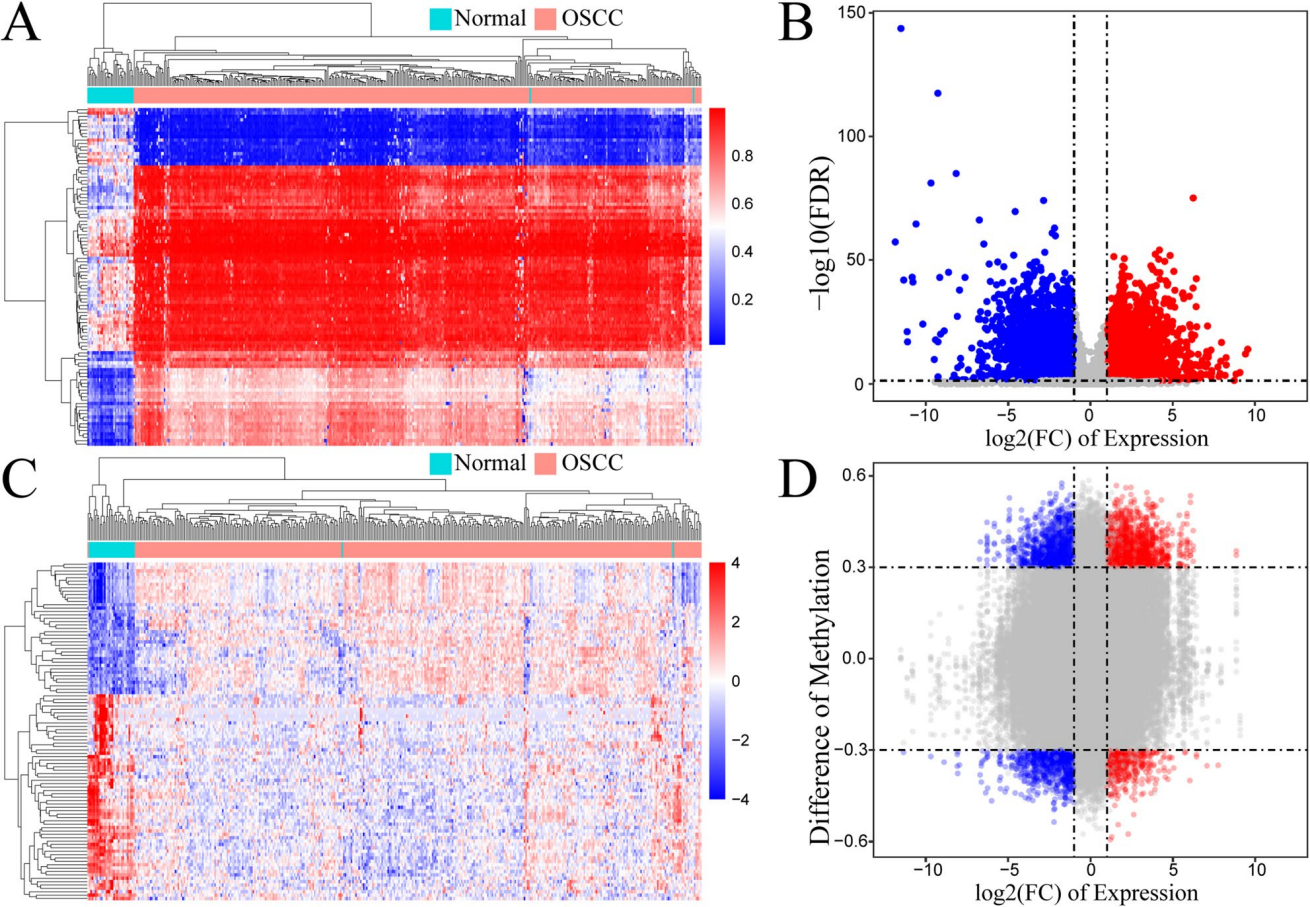
Alterations of transcriptome and methylome profiling in oral squamous cell carcinoma (OSCC) samples. (**A**) Unsupervised hierarchical clustering of normal and OSCC samples using the top 100 differentially expressed gene (DEG) probes according to F score. The heatmap shows DEGs arranged in rows (upregulation colored in red and downregulation in dark blue) and samples in columns (OSCC samples colored in pink and adjacent normal samples in turquoise). (**B**) Volcano plot of DEGs using the cutoff of |logFC| ≥ and the padj < 0.05. LogFC  ≤ were downregulated DEGs colored in blue and logFC ≥ were upregulated DEGs colored in red. (**C**) Unsupervised hierarchical clustering of normal and OSCC samples using the top 100 differentially methylated probes according to F score. The heatmap shows differentially methylated CpGs (DMCpGs) arranged in rows (hypermethylation colored in red and hypomethylation in dark blue) and samples in columns (OSCC samples colored in pink and adjacent normal samples in turquoise). (**D**) Scatter plot of DMCpGs and related DEGs using the cutoff of |logFC| ≥ 1, |delta of Beta| ≥ 0.3, and the padj < 0.05. LogFC  ≤  1 were downregulated DEGs colored in blue and logFC  ≥  1 were upregulated DEGs colored in red. The delta of Beta  ≥  0.3 were hypermethylated DEGs in the upper region and delta of Beta ≤ 3 were hypomethylated DEGs in the lower region. This heatmaps was generated by the R (version 4.1.0, https://www.r-project.org).

A flowchart of the study is shown in Fig. 1. A total of 8074 DEGs and 10,084 DMCpGs were identified. Clustering based on the DEGs or DMCpGs revealed two distinctive sample clusters, indicating the possibility to distinguish between OSCC samples and adjacent tissue samples (Fig. 2A,B). Of the 8074 DEGs, 4327 were upregulated and 3747 were downregulated (Fig. 2C). Of the 10,084 DMCpGs, 5937 were hypermethylated DMCpGs (HyperCpGs) and 4147 were hypomethylated DMCpGs (HypoCpGs). Both HyperCpGs and HypoCpGs were correlated with the upregulation and downregulation of genes (Fig. 2D). These results indicate that the regulation of gene expression by methylation may be complex and multimodal.

### Relationship between distribution pattern of methylation alterations and gene expression

**Figure 3:**
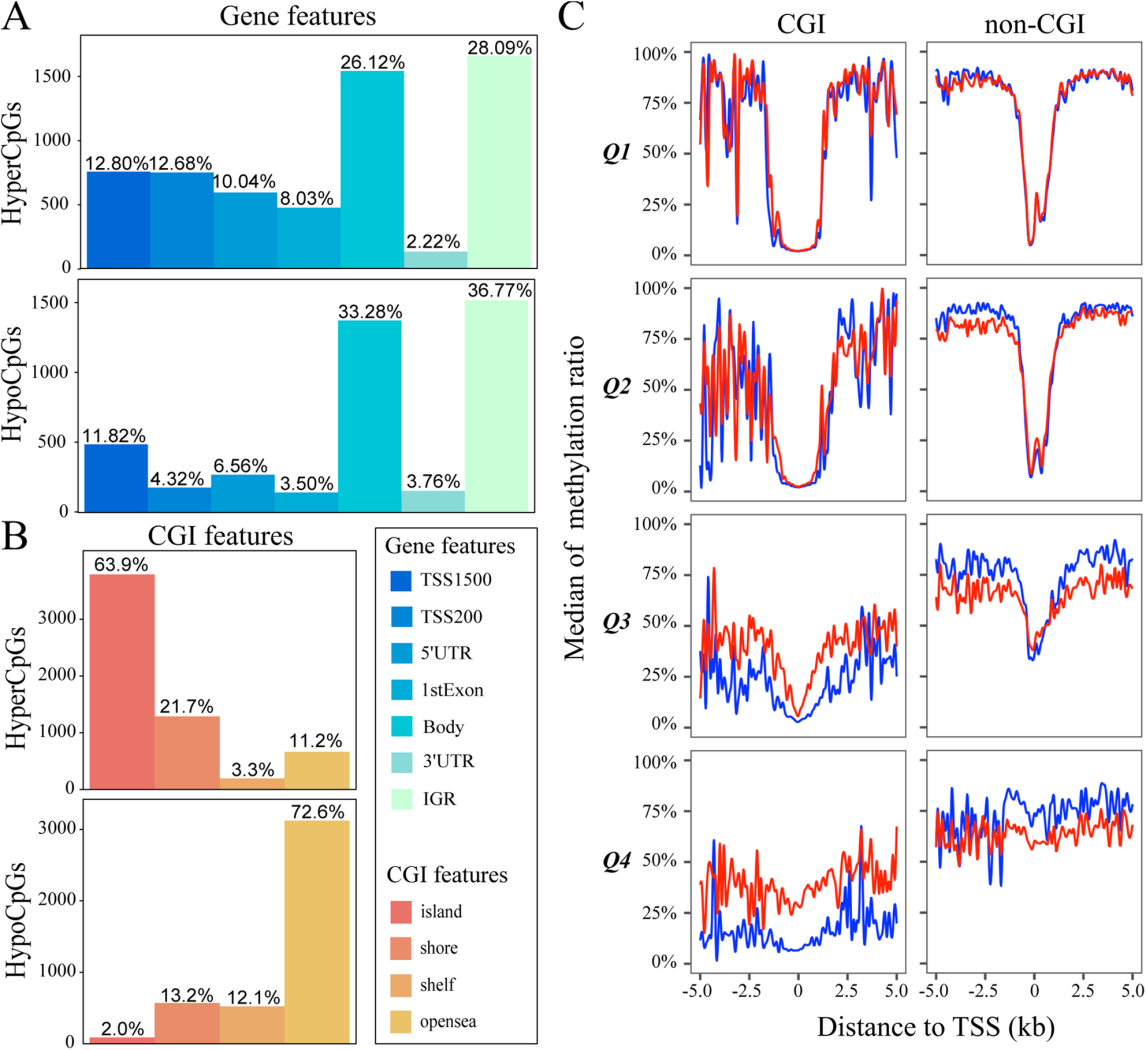
Complex pattern of DNA methylation in oral squamous cell carcinoma (OSCC) samples. (**A**) Bar plots of numbers and ratios of hypermethylated and hypomethylated CpGs grouped by gene features. The gene features colored in cold tunes were divided into regions 200 bp upstream of transcriptional start site (TSS200), 1500 bp upstream of TSS (TSS1500), 5′-untranslated regions (5′-UTR), 3′-UTR, the first exon (1st exon), and gene body and intergenic regions (IGR). (**B**) Bar plots of numbers and ratios of hypermethylated and hypomethylated CpGs grouped by gene and CpG island (CGI) features. The CGI features were divided according to the distance to CGI, including island, shore (regions within 2 kb upstream or downstream of island), shelf (regions of 2–4 kb upstream or downstream of island), and open sea (regions outside of island, shore, and shelf). (**C**) Plots of the median methylation values per 100 bp distance grouped by gene expression quartiles based on the expression levels in either tumor or normal samples (n = 5493, 5493, 5493, and 5496 for Q1, Q2, Q3, and Q4, respectively; Q4 is the highest expression) showing methylation ratio at 100 bp segments including genomic loci within and outside CpG islands in genes with promoter associated CpG islands. The curves were colored in red in OSCC samples and in blue in normal samples.

DMCpGs across different genomic regions were not randomly distributed across the genome. Most DMCpGs are found in the gene body and intergenic regions (IGR). HyperCpGs had a higher proportion located 200 bp upstream of transcriptional start sites (TSS200), 5′-untranslated regions (UTR), and 1st exon, whereas HypoCpGs were located in the gene body and intergenic regions (Fig. 3A). The results indicated that there was a high density of methylated CpGs in several kb regions upstream and downstream of the transcriptional start sites (TSS). The distribution pattern around the CGI differed significantly between HyperCpGs and HypoCpGs. The distribution of HyperCpGs was significantly enriched within CGI (62.9%), whereas HypoCpGs were mostly enriched in the open sea regions (72.6%) (Fig. 3B), indicating that CpG methylation in CGI and non-CGI was potentially functional in gene expression. To further integrate the distribution pattern around TSS, CGI, and gene expression, we plotted the CGI and non-CGI methylation levels of every gene within four expression quartiles grouped by distance to the TSS (Figs. 3C and S1). Then, to check the potential bias owning to unpaired samples, we divided the samples into paired normal samples (pNormal), paired Tumor samples (pTumor) and unpaired single Tumor samples (sTumor) and found that the methylation pattern of pTumor was similar to that of sTumor instead of pNormal, showing the robustness of results (Fig. S2). HypoCpGs proximal to the TSS (approximately ± 1 kb) were observed in both OSCC and adjacent samples in the highly expressed CGI genes. CGI CpGs with low gene expression levels exhibited a higher number of HyperCpGs around TSSs in OSCC samples than in adjacent tissues, indicating that hypermethylation-induced silencing of tumor suppressor genes was more evident in transcriptionally silent genes with CGI. Moreover, HypoCpGs were observed in non-CGI CpGs with low gene expression and in those with high gene expression away from the TSS, suggesting a potential role of non-CGI HypoCpGs in the regulation of oncogenes and tumor-suppressor genes.

### Correlation between methylation levels and gene expressions

**Figure 4:**
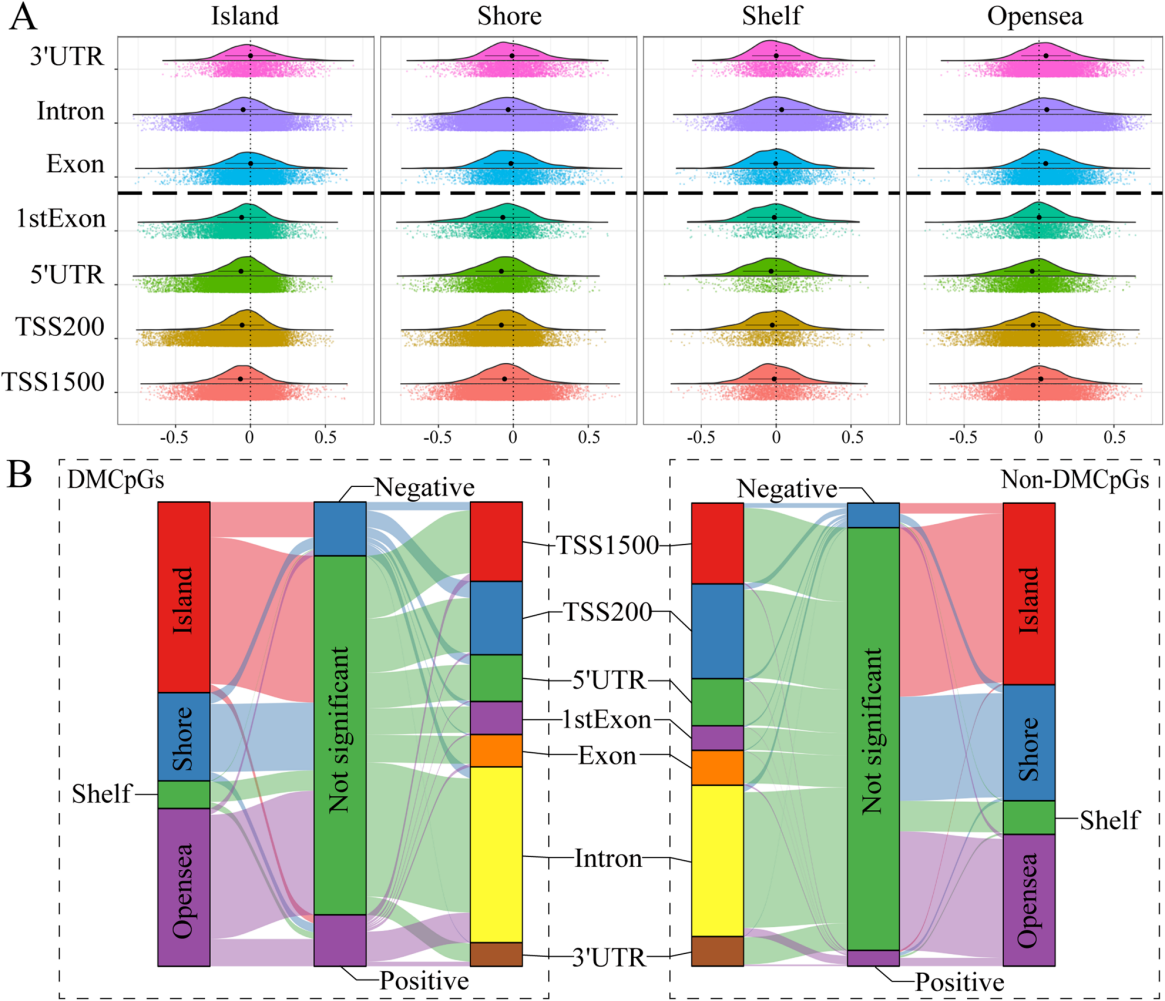
Complex links between DNA methylation and gene expression. (**A**) Scatter plot of correlation coefficient between DNA methylation and gene expression faceted by gene and CpG island (CGI) features. The points on the left of “coefficient = 0” line denoted upregulated or downregulated gene expression with hypermethylation or hypomethylation, respectively, while those on the right denoted upregulated or downregulated gene expression with hypomethylation or hypermethylation, respectively. The mean correlation values and 95% confidence intervals were shown. The horizontal bold dotted line separated the rows into non-promoter (upper) and promoter rows (lower). (**B**) Sankey diagram of the distribution of correlations between DNA methylation and gene expression in differentially methylated CpGs (DMCpGs) and non-DMCpGs according to gene and CGI features. The correlations were separated into significantly positive, significantly negative, and non-significant correlations according to the cutoff of false discovery rate < 0.05 and |correlation value|> 0.3. The height of rectangles was proportional to the distribution ratio in the region. The degree of connecting line thickness was proportional to number in this type of correlation.

We recognized that HyperCpGs and HypoCpGs might function differently in genes with CGI and non-CGI CpGs in OSCC samples. To further understand the correlations between CpG alterations and gene expression, we defined CpGs as positively-correlated CpGs (PosCpGs), wherein HyperCpGs and HypoCpGs were positively correlated with upregulation and downregulation of gene expression, respectively, and vice versa, as negatively-correlated CpGs (NegCpGs). We plotted the correlation between methylation and gene expression levels grouped by the genomic distribution of methylated CpGs. NegCpGs were highly concentrated around CGIs and within the promoter, while PosCpGs were highly distributed within the gene body, and the majority of CpGs were open sea (Fig. 4A). The correlation modes were gradient from CGI to the open sea. The results indicated that the correlation between methylation and gene expression levels was diverse but organized across the genomic distribution. However, not all correlations between methylation and gene expression levels were statistically significant. We further divided the correlation into significantly positive, significantly negative, and non-significant. Significant associations were observed more frequently in the DMCpGs than in the non-DMCpGs. CGI CpGs located in the promoter tended to have significantly negative associations with gene expression, whereas CpGs away from CGI and located in the gene body had significantly positive associations (Fig. 4B).

### Screening of prognostic DEGs with significant methylation correlation

**Figure 5:**
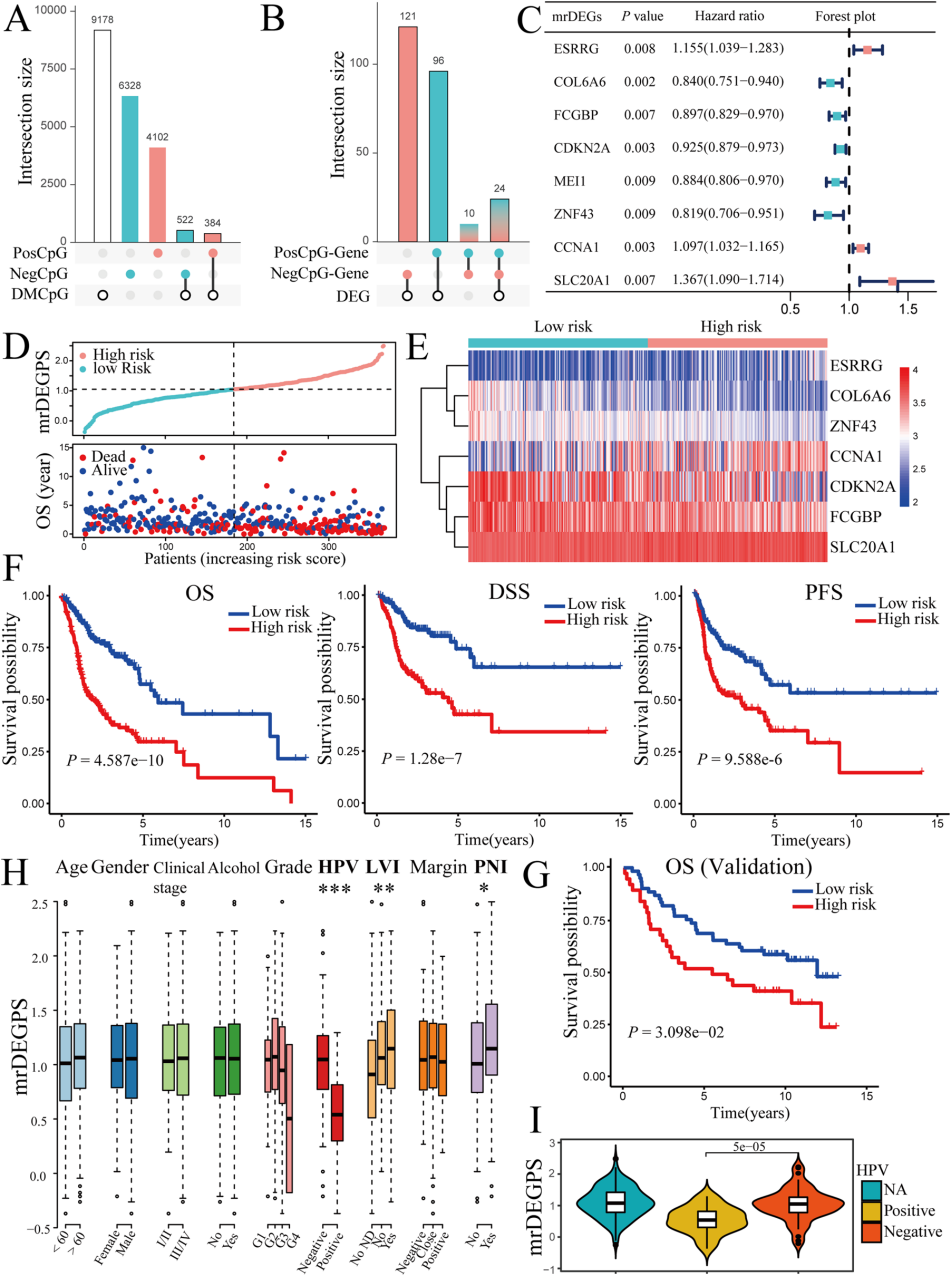
Development and validation of prognostic methylation-related differentially expressed genes (mrDEGPS) model in oral squamous cell carcinoma (OSCC) samples. (**A**) UpSet plot of number of positively correlated CpGs (PosCpGs) (turquoise), negatively correlated CpGs (NegCpGs) (pink), and DMCpGs (black border) in OSCC samples. The intersections of differentially methylated CpGs (DMCpGs) with PosCpGs or NegCpGs were expressed using turquoise or pink bars with black border. (**B**) UpSet plot of number of genes with PosCpGs (PosCpG-Gene, turquoise), NegCpGs (NegCpG-Gene, pink), and DEGs (black border) in OSCC samples. The intersections of DEGs with PosCpG-Genes or NegCpG-Genes were expressed using turquoise or pink bars with black border. The intersections between PosCpG-Genes and NegCpG-Genes were expressed using graduating color from turquoise to pink. (**C**) Partial results of multivariate Cox proportional hazards regression analysis of mrDEGs using the cutoff of *P* < 0.01. MrDEGs were defined as DEGs with posCpG, NegCpG, or both. The vertical dotted line was the invalid line of HR = 1. The mrDEGs located on the left and right of the invalid line were prognosis-beneficial and prognosis-harmful mrDEGs colored in turquoise and pink, respectively. (**D**) MrDEGPS and overall survival (OS) in patients sequenced by increasing mrDEGPS. The low- and high-risk patients were divided by the cutoff of mrDEGPS = 1.0256. OS was calculated based on the follow-up time, if patients survived (blue), or time until death (red). (**E**) Heatmap of high- and low-risk OSCC samples grouped by mrDEGPS according to the simulation result of mrDEGPS model. The heatmap shows mrDEGs arranged in rows (upregulation colored in red and downregulation in dark blue) and samples in columns (high-risk OSCC samples colored in pink and low-risk OSCC samples in turquoise). (**F**) Kaplan–Meier plots for OS, disease-specific survival, and progression-free survival grouped by low- and high-risk patients in the training set (The Cancer Genome Atlas (TCGA) dataset)). (**G**) Kaplan–Meier plots for OS grouped by low- and high-risk patients in a validation set (GSE41613). (**H**) Box plots of mrDEGPS grouped by different clinical features including age, gender, clinical stage, alcohol habit, histopathological grade, HPV infection, lymphovascular invasion, surgical margin, and perineural invasion in the training set (TCGA dataset). *, **, and ***indicate *P* < 0.05, *P* < 0.01, and *P* < 0.001, respectively. (I) Violin plot of mrDEGPS grouped by HPV (+), HPV (−), and HPV status not available (NA) in a validation set (GSE87053).

To further screen the CpGs significantly correlated with gene expression, we intersected DMCpGs with PosCpGs or NegCpGs and obtained 522 and 384 DMCpGs that exhibited significant positive and negative correlations with gene expression, respectively (Fig. 5A). We then intersected DEGs with genes containing selected PosCpGs or NegCpGs and found 121 DEGs with positively related DMCpGs, 96 DEGs with negatively related DMCpGs, and 24 DEGs with both positively and negatively related DMCpGs (Fig. 5B). The intersection results further indicate the complexity of methylation regulation. It was difficult to predict the final gene expression, particularly when multiple methylation sites were altered. Here, we defined DEGs with significantly correlated DMCpGs as methylation related DEGs (mrDEGs). To determine the prognostic value of the 241 mrDEGs in OSCC, a multivariate Cox regression analysis was performed with a cutoff of *P* < 0.01. We found that three mrDEGs (*ESRRG*, *CCNA1*, and *SLC20A1*) were associated with a poor prognosis and significantly increased hazard ratio (HR), whereas five mrDEGs (*COL6A6*, *FCGBP*, *CDKN2A*, *MEI1,* and *ZNF43*) served as protective genes with HR < 1 (Fig. 5C). However, *MEI1* did not reach a significant level in the Least Absolute Shrinkage and Selection Operator (LASSO) and was thus abandoned.

### Establishment and validation of the prognostic signature

We then built an mrDEGs predictive signature (mrDEGPS) using seven survival-relevant mrDEGs. The mrDEGPS for each patient was calculated using the following formula:

$$ \begin{aligned} {\text{mrDEGPS}} & = { }0.248{\text{exp}}\left( {ESRRG} \right){ } + { }0.047{\text{exp}}\left( {CCNA1} \right){ } + { }0.200{\text{exp}}\left( {SLC20A1} \right) \\ & \quad - \;0.138{\text{exp}}\left( {COL6A6} \right){ } - { }0.0507{\text{exp}}\left( {FCGBP} \right){ } - { }0.045{\text{exp}}\left( {CDKN2A} \right){ } - { }0.161{\text{exp}}\left( {ZNF43} \right) \\ \end{aligned} $$

We divided the patients with OSCC into low- and high-risk groups (Fig. 5D). The expressions of *ESRRG*, *CCNA1,* and *SLC20A1* were comparatively higher in the high-risk group than in the other groups (Fig. 5E). Prognosis comparison showed that low-risk patients had significantly higher overall survival (OS) (*P* = 4.587e−10), disease-specific survival (DSS) (*P* = 1.28e−07), and progression-free survival (PFS) (*P* = 9.588e−6) than that in high-risk patients (Fig. 5F). The validation analysis in a small sample size (n = 97) demonstrated that patients in the high-risk group had poorer overall survival (*P* = 3.098e−2) than those patients in the low-risk group (Fig. 5G). The area under the curve (AUC) of 1-year survival was 0.714 and 0.702 in the training and validation datasets, respectively. Compared with the clinical stage, mrDEGPS displayed superior predictive performance.

### Association between mrDEGPS with clinical features and human papillomavirus (HPV)

Next, we explored the association between the mrDEGPS and clinical features (Fig. 5H). We found that the mrDEGPS did not differ between patients with different age groups, gender, alcohol consumption, and surgical margin status (*P* > 0.05). Notably, there was no difference in mrDEGPS in the different clinical stages and histopathological grades (*P* > 0.05), implying the absence of linear correlation between mrDEGPS and these traditional survival stratification factors; thus, mrDEGPS was a potentially independent prognostic factor. We also found that the mrDEGPS was significantly associated with lymphovascular and perineural invasion. As both lymphovascular and perineural invasion were independent prognostic factors^24^, this result was consistent with mrDEGPS as a prognostic factor. Moreover, the human papillomavirus (HPV) infection was associated with higher mrDEGPS (*P* < 0.001), indicating that HPV might partially promote epigenetic alterations. We further included patients with HPV status not available (NA) in the analysis and found no differences in mrDEGPS between HPV (NA), HPV ( +), or HPV (−) (Fig. 5I), which indicated that the mixed population neutralized the difference between HPV ( +) and HPV (−).

### Association between mrDEGPS and TIME

**Figure 6:**
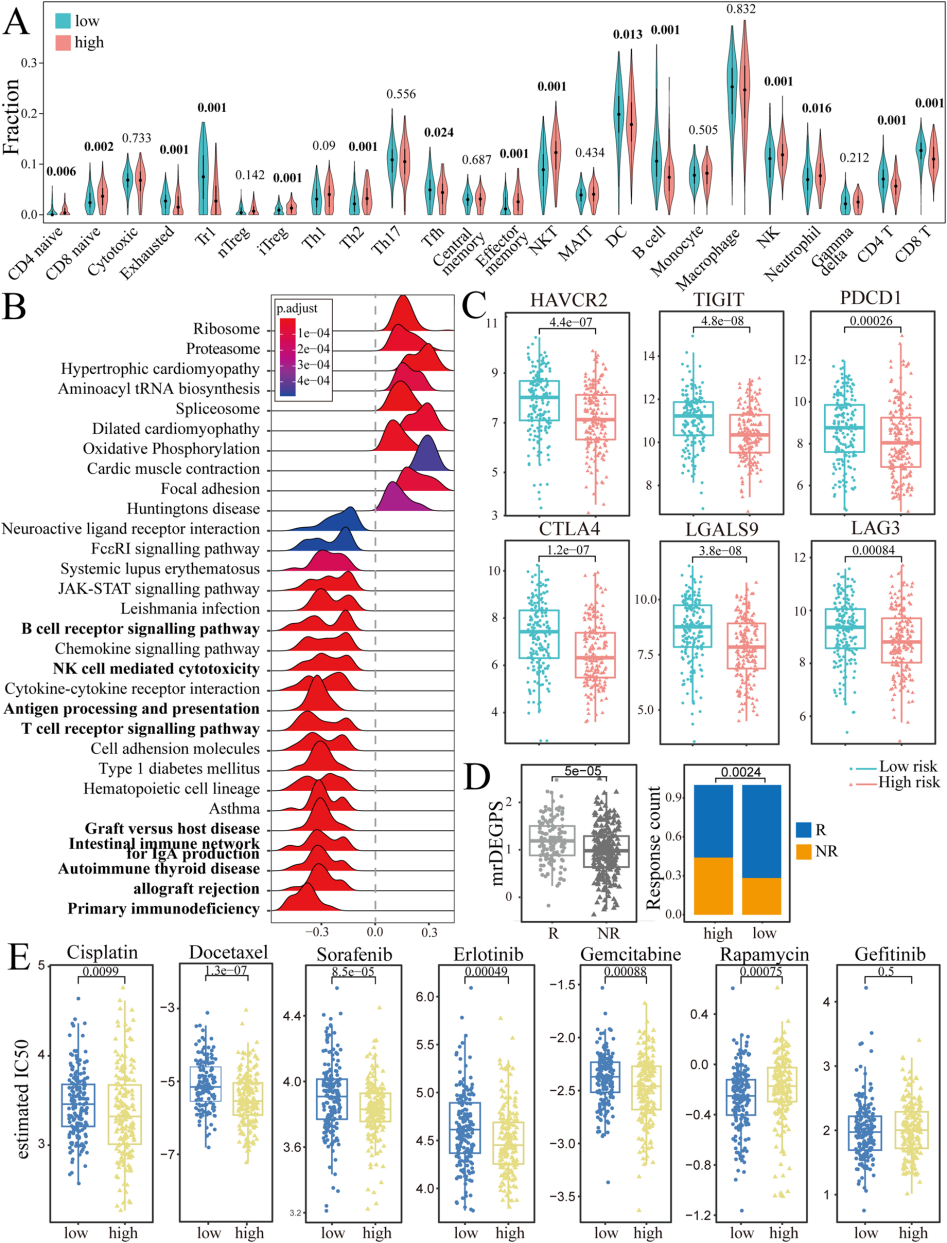
Methylation-related differentially expressed genes (mrDEGPS) model for the prediction of immunotherapy and chemotherapy in patients with oral squamous cell carcinoma (OSCC). (**A**) Violin plots of levels of 24 types of immune cells grouped by low- and high-risk samples. (**B**) Gene set enrichment analysis (GSEA) results of genes related to mrDEGPS. Significant enrichments in immune-related Kyoto Encyclopedia of Genes and Genomes (KEGG)^26^ pathways were labeled in bold. GSEA was performed to enrich the KEGG pathways in gene relation value with mrDEGPS. A false discovery rate less than 0.05 and an absolute value of the enrichment score greater than 0.5 were defined as the cutoff criteria. (**C**) Box plots of immune-checkpoint-relevant gene expressions including CTLA-4, LAG-3, GAL9, PD-1, PD-1LG2, PD-L1, TIM-3, and TIGIT. (**D**) The mrDEGPS grouped by immunotherapeutic response and the distribution of immunotherapeutic response in low- and high-risk groups stratified by mrDEGPS. (**E**) Differential chemotherapeutic responses in high- and low-risk patients to chemotherapeutic drugs, including cisplatin, docetaxel, sorafenib, erlotinib, gemcitabine, rapamycin, and gefitinib. The predictions for other significantly differential chemotherapeutic responses are provided in the Supplementary Information. Tc, cytotoxic T cells; Tex, exhausted T cells; Tr1, type 1 regulatory T cells; nTreg, natural regulatory T cells; iTreg, induced regulatory T cells; Th1, T-helper 1 cells; Th2, T-helper 2 cells; Th17, T-helper 17 cells; Tfh, follicular T-helper cells; Tcm, central memory T cells; Tem, effector memory T cells; NKT, natural killer T cells; MAIT, mucosal-associated invariant T cells; NK, natural killer cells; DC, dendritic cells.

Since the selected mrDEGs were relevant to the immune response, we hypothesized that mrDEGPS might have the capacity to identify alterations in TIME. The results showed that the high-risk group samples had reduced proportions of exhausted T cells, type 1 regulatory T cells, follicular T-helper cells, dendritic cells, B cells, CD4^+^ T cells, and CD8^+^ T cells, and increased proportions of naive CD4^+^ T cells, naïve CD8^+^ T cells, induced regulatory T cells, T-helper 2 cells, effector memory T cells, natural killer T cells, natural killer cells, and neutrophils. (Figs. 6A and S3). It has been reported that DCs, CD8^+^ T cells, and CD4^+^ T cells display a beneficial effect on survival, while neutrophils, NK cells, and Tem cells display harmful effects in breast cancer^25^. Our results implied that high-risk patients might have an altered survival–harmful TIME. To further characterize the potential signaling pathways involved in the influence of mrDEGPS, gene set enrichment analysis (GSEA) was performed to enrich the Kyoto Encyclopedia of Genes and Genomes^26^ pathways ranked by gene correlation values with mrDEGPS. Half of the enriched pathways were associated with immune processes like “primary immunodeficiency,” “allograft rejection,” “autoimmune thyroid disease,” “intestinal immune network for IgA production,” “T cell receptor signaling pathway,” “antigen processing and presentation,” “natural killer cell-mediated cytotoxicity,” and “B cell receptor signaling pathway” (adi. *P* < 0.01) (Fig. 6B).

### Prediction of immunotherapy and chemotherapy by mrDEGPS

**Figure 7:**
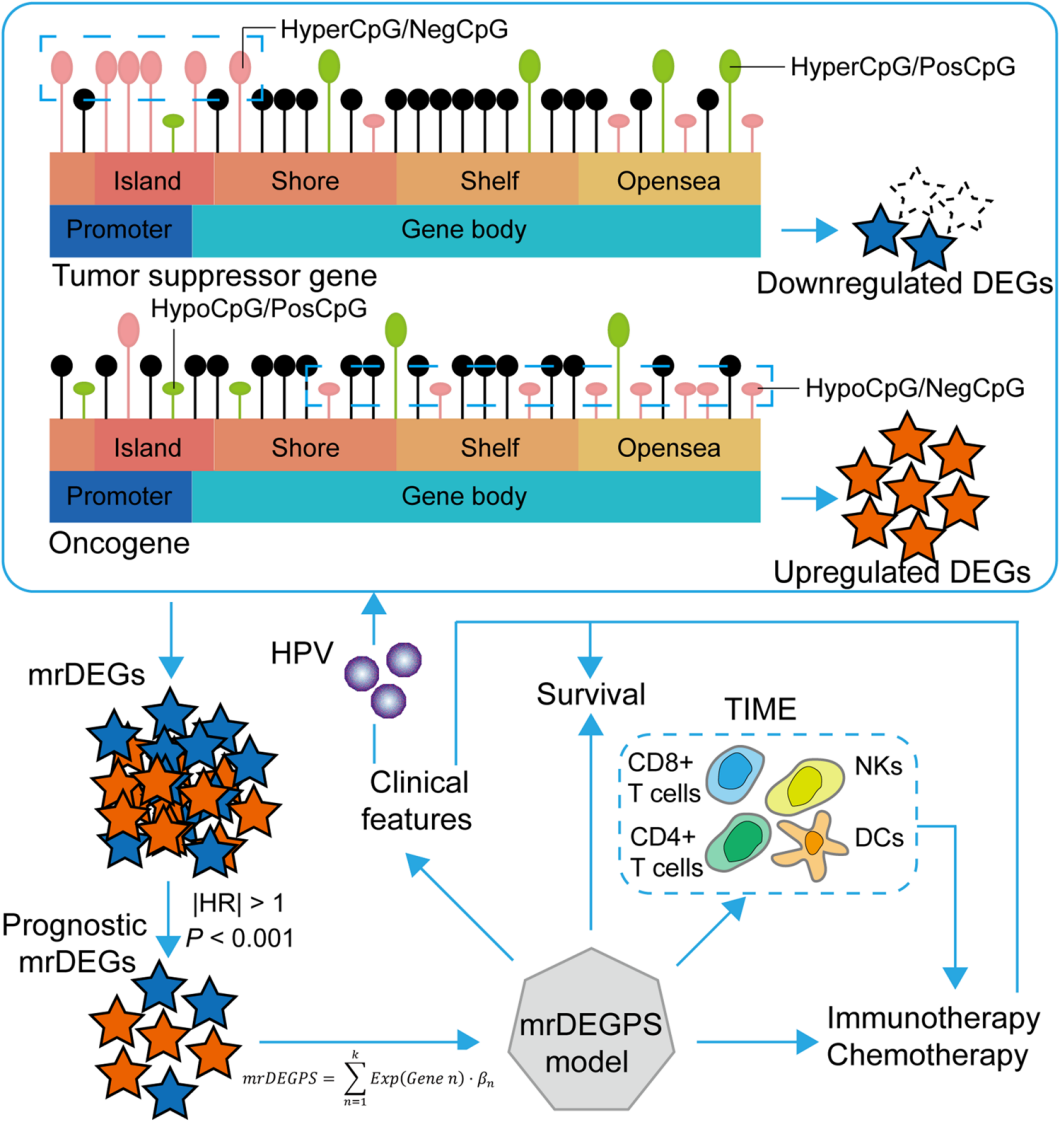
Illustration of findings in the study. The black lollypop indicates CpGs without significant changes. The dotted blue frame indicates the potential alterations in tumor suppressor genes and oncogenes in oral squamous cell carcinoma.

T-cell receptor signaling is the essential basis for immunotherapy and may participate in chemotherapy resistance^27,28^. Current immunotherapy is mainly achieved by antibody blocking of *CTLA-4* or *PD-1* pathway^29^. Studies indicate that *LAG3*, *LGALS9*, *HAVCR2,* and *TIGHT* could be the next-generation immunotherapy checkpoints^30–33^. We found that all these immunotherapy checkpoints were downregulated in high-risk patients (*P* < 0.001) (Fig. 6C). However, there was no significant difference in *PD-L1*, indicating that mrDEGPS was correlated with TIME rather than with tumor cells (Fig. S4). The mrDEGPS was higher in patients with a response (*P* = 0.00005) and the response rate was significantly lower in high-risk patients (*P* = 0.0024) (Fig. 6D). These results indicate that mrDEGPS low-risk patients might benefit from immunotherapy. For chemotherapy, the estimated half-maximal inhibitory concentration difference between low- and high-risk patients was inconsistent among drugs, which was not significant for gefitinib, lower in low-risk patients for rapamycin, and lower in high-risk patients for cisplatin, docetaxel, sorafenib, erlotinib, and gemcitabine (Figs. 6E and S5). We observed that the result might provide a reference for chemotherapeutic drug selection for individuals, and thus facilitated the survival of patients with OSCC. In summary, mrDEGPS screened from complex transcriptome–methylome interactions could facilitate the prediction of survival and immunotherapeutic efficacy in patients with OSCC (Fig. 7).

## Discussion

Several studies have demonstrated a strong relationship between epigenetic and genetic aberrations in tumorigenesis^34^. It is commonly believed that epigenetic changes, such as DNA methylation, can drive abnormal gene expression of crucial genes involved in the development and progression of cancer, including head and neck cancer^35^. Hypermethylation of tumor suppressor genes and hypomethylation of proto-oncogenes at the promoter sites are associated with carcinogenesis and progression of OSCC^36,37^. For example, several studies have suggested that hypermethylation of *PAX1* and *ZNF582* genes is associated with aggressive progression and poor survival^38–40^.

Although the effect of promoter methylation changes has been studied extensively, increasing evidence from genome-wide methylome studies suggests that the methylation patterns are complex and cancer-type-specific^41–43^. The term “CpG island methylator phenotype” has been repeatedly used over decades to describe widespread CpG island promoter methylation^44,45^. However, only around 4–8% CGIs exhibit tissue-specific methylation, while approximately 70% of annotated gene promoters are associated with a specific CGI^46^. Therefore, there must be undisclosed cancer-type-specific methylation outside CGIs. Our results showed that CpGs away from islands, denoted as open sea CpGs, may facilitate gene expression of oncogenes in OSCC.

Despite CGI features, cumulative evidence indicates that the transcriptome–methylome interaction is not restricted to promoters and TSS^47^. In contrast to the repression of promoter methylation on expression, gene body methylation orchestrates transcription in a complex pattern, which is in contrast to the repression of promoter methylation. Approximately half of CGIs in mammalian genomes are not associated with a known gene promoter and are referred to as orphan CGIs^48^. Orphan CGIs that do not map to promoters of any protein-coding or non-coding transcripts but possess chromatin and transcriptional markers may reflect enhancer activity^49^. Orphan CGIs display most of the evolutionarily conserved methylation differences among tissues, indicating their possible role in tissue specification^50^. Our results indicated that CpGs located in the gene body might have a positive association with gene expression, suggesting that most tissue-specific methylation CGIs are not located at promoter regions in OSCC^46^. These findings may potentially facilitate research on aberrant methylation of these rarely investigated regions.

Another hypothesis regarding the non-promoter DMCpGs is that most genes have two or more TSSs; therefore downstream TSS are probably within the bodies of the transcriptional units of the alternative upstream promoters^51^. A large-scale full-length cDNA analysis to explore the budding yeast transcriptome showed that alternative promoters could be located at CGIs or non-CGIs, and combinations of an upstream non-CGI and a downstream CGI, or vice versa, may also occur. It is well known that alternative promoters contribute to context-specific isoform expression and the regulation of isoform diversity^52^. However, it is difficult to interpret the link between expression and methylation in genes with multiple TSSs. Probes that are used to measure expression detect the output of all alternative promoters; however, only a single isoform might be dominant in OSCC cells. Methylation of a downstream promoter blocks transcription from that promoter, which would allow the elongation of a transcript emanating from an upstream promoter, thereby leading to an apparent discordance between methylation and expression.

Currently, most methylome studies use the term “methylation-driven gene”^53,54^. However, we believe that the expression “driven” may be misleading. Considering the complex methylation patterns of alternative promoters, we conservatively referred to methylation-driven DEGs as mrDEGs. The mrDEGs were simultaneously hypermethylated and hypomethylated at different sites. There is a high possibility that CGI HyperCpGs function synergistically with open sea HypoCpGs. However, due to the limitation of the technique, it was challenging to deduce whether the gene expression difference was the counterbalance effect of antagonistic methylation alterations. Therefore, mrDEGs may be a suitable terminology for DEGs with differentiated methylation.

The mrDEGPS model was established using seven prognostic mrDEGs (*ESRRG*, *CCNA1*, *SLC20A1*, *COL6A6*, *FCGBP*, *CDKN2A,* and *ZNF43*). Methylation of *ESRRG* and *CCNA* has been investigated in head and neck cancers and is associated with poor prognosis^55–57^. The prognostic significance of *CDKN2A* and *ZNF43* promoter hypermethylation has also been confirmed in colorectal cancers^58,59^. To the best of our knowledge, polymorphisms of *COL6A6* and *SLC201A* have been preliminarily investigated in other diseases, and their methylation, possibly related to the polymorphism, is unknown^60,61^.

In our study, we found that mrDEGPS could serve as an independent prognostic predictor. MrDEGs, including *FCGBP*, *COL6A6*, *CDKN2A,* and *CCNA1* correlated with immune cell response, T-cell phenotype modulation, and treatment response to doxorubicin^62–65^, whereas *ESRRG* expression was negatively correlated with CD4^+^ T cell activation. The role of mrDEGPS in TIME, immunotherapy, and chemotherapy responses was further investigated. Activated immune cell clusters, including CD8^+^ effector cells, exhibited lower infiltration in high-risk OSCC samples, indicating that the mechanism of prognostic function of mrDEGPS might be attributed to TIME alterations.

In addition, HPV may significantly affect the methylation levels of prognostic mrDEGs. Differences in methylation in HPV-driven cases were revealed in a previous study of the OSCC epigenetic landscape^66^. For example, DNA methylation of *IDO1* in head and neck squamous cell carcinomas (HNSC) correlates with HPV status and survival^67^. Methylation markers for diagnosis of OSCC were independent of HPV infection^68^. It could be speculated that HPV correlated with partially methylated gene expression, whereas mrDEGs were fitted in the prognosis model rather than a diagnosis model. However, it is paradoxical that low-risk samples have a higher HPV infection rate since HPV is a well-known poor prognostic factor. Further studies are required to elucidate the role of HPV in DNA methylation.

However, this study had some limitations. Firstly, the validation set was limited. However, considering the cancer-specific property of epigenetic modulation, it might not be reasonable to validate the model in other datasets (for example, a dataset of HNSC included samples other than OSCC). Second, the prediction reliability of the score model was not verified in our clinical observations owing to limited resources. In addition, the cohorts lacked partial clinicopathological data, and ethnic differences existed among the groups. However, we collected the best available matched data. These results may be further confirmed by genomic big data. As mrDEGPS is a prognostic model irrespective of the clinical stage, an improved novel staging system could be explored by combining genomic and clinical risk factors. Third, this study selected adjacent normal tissue as control. The control may be affected by field cancerization, leading to phenotypes different from true healthy tissue. However, we could not change the TCGA and other dataset as authors of secondary analysis. Lastly, immunotherapy and chemotherapy were more frequently prescribed for patients with late-stage OSCC. Further validation should be performed using data from patients with stage III/IV disease. Big data has evolved as the ubiquitous watchword of medical innovation^69^. We believe that the prevalence of medical big data can mitigate these limitations.

In conclusion, the transcriptome–methylome interaction pattern in OSCC is complex. Moreover, mrDEGPS could predict patient survival and responses to immunotherapy and chemotherapy and facilitate clinical decision-making in patients with OSCC.

## Materials and methods

### Data acquisition and preprocessing

We downloaded DNA methylation (528 HNSC samples and 50 normal samples), RNA sequencing (500 HNSC samples and 44 normal samples), and clinical information data from The Cancer Genome Atlas (TCGA) HNSC cohort from UCSC-Xena (https://xenabrowser.net/) on August 28, 2019. Excluding non-OSCC samples and samples with unavailable methylome or transcriptome data, a TCGA-OSCC cohort of 391 OSCC samples and 41 adjacent normal samples was enrolled in this analysis. We downloaded GSE41613, comprising 96 OSCC samples, from the Gene Expression Omnibus (GEO) database (https://www.ncbi.nlm.nih.gov/geo/). All data were normalized in the R computing environment (version 4.1.0) using the DESeq2 or limma packages (https://www.r-project.org). The data were utilized according to the data access policies of TCGA and GEO. All the studies were performed in accordance with the Declaration of Helsinki.

### Analysis of DNA methylation pattern in OSCC and its regulation on mRNA transcription

Gene expression in TCGA-OSCC cohort was correlated with the normalized beta value of HM450K probes using Spearman’s rank correlation followed by false discovery rate (FDR) correction using the FDR method. Methylation-gene relations with |correlation value|> 0.3 and FDR < 0.05 were considered significant, and kernel density estimation was used to plot the distribution of significant probes around the TSS ± 5 kb. The methylation level of each probe was measured by the beta value, which ranged from 0 to 1 (representing unmethylated to fully methylated levels, respectively). Similarly, we removed probes with missing beta values in ≥ 5% samples. The remaining probes with missing beta values were imputed using the k-nearest neighbors algorithm.

We mapped the probes to the promoter regions of the genes, which were defined as − 1.5 to 0 kb regions around the TSS. Next, the DNA methylation level of a gene was defined as the average beta value of the probes that mapped to its promoter region. Finally, samples with paired mRNA expression and DNA methylation profiles were analyzed, which involved 17,481 genes and 384 samples, to determine DMCpGs and DEGs. The Spearman rank correlation of CpG methylation levels with related gene expression was analyzed, and significant associations were based on the criteria of |correlation value|> 0.3 and FDR < 0.05.

### Identification and validation of mrDEGPS

Kaplan–Meier analysis was used to evaluate the relationship between the genes and survival of patients with OSCC. The LASSO binary logistic regression model and multivariate Cox regression were adopted after primary filtration. The linear combination of the regression coefficient β derived from the multivariate Cox regression model and multiplied by the corresponding mRNA levels generated a prognostic signature. The mrDEGPS for each patient was calculated using the following risk score formula:

$$ {\rm mrDEGPS} = \mathop \sum \limits_{n = 1}^{k} {\rm Exp}\left( {{\rm Gene} \,n} \right)\cdot\beta_{n} $$

Patients were divided into high- and low-risk groups by setting the median risk score as the cut-off value. The OS, DSS, and PFS of the two groups were calculated using the Kaplan–Meier method with the log-rank test. Receiver operating characteristic (ROC) curves were generated to assess the predictive performance of the prognostic model. The expression patterns of genes in this prognostic model were visualized using the “pheatmap” package. In the validation analysis, we verified the Kaplan–Meier plot and ROC test in GEO using another OSCC cohort, GSE41613. The clinical features of the patients with low or high mrDEGPS were analyzed and compared.

### Analysis of correlation between the survival risk model and tumor immune infiltration

ImmuCellAI (http://bioinfo.life.hust.edu.cn/ImmuCellAI#!/) was utilized to analyze the fraction of 24 types of immune cells in high- and low-risk samples with OSCC^70^. These immune cells included 18 subtypes of T cells, namely CD4^+^ T cells; CD8^+^ T cells; naive CD4^+^ T cells; naive CD8^+^ T cells; cytotoxic T cells; exhausted T cells; type 1 regulatory T cells; natural regulatory T cells; induced regulatory T cells; T-helper 1, 2, and 17 cells; follicular T-helper cells; central memory T cells; effector memory T cells; natural killer T cells; mucosal-associated invariant T cells; and gamma-delta T cells, as well as six other types of immune cells including B cells, natural killer cells, monocytes, macrophages, neutrophils, and dendritic cells. Immune Cell Abundance Identifier (ImmuCellAI) built an immune cell‐based support vector machine model for the prediction of immunotherapy response (AUC: 0.80–0.91), and the model was used to estimate the response results of OSCC.

GSEA was performed using the R package “clusterprofiler” to determine the enrichment of previously defined biological processes in the ranked correlated gene with risk score using RNA-seq data from TCGA-OSCC cohort. The raw count data of gene expression from the TCGA-OSCC cohort were normalized using the variance stabilizing transformation function in R package “DESeq2,” and then submitted to ImmuCellAI (http://bioinfo.life.hust.edu.cn/web/ImmuCellAI/) to estimate the abundance of immune cells, particularly the T-cell proportions, and to predict the response of immune-checkpoint inhibitor treatments.

## Supplementary Information

Supplementary Information.

## Data Availability

The datasets analysed during the current study are available in the The Cancer Genome Atlas (TCGA) Head and Neck Squamous Cell Carcinoma (HNSC) cohort from UCSC-Xena (https://xenabrowser.net/) dated August 28th, 2019, and a Gene Expression Omnibus dataset (GSE41613, https://www.ncbi.nlm.nih.gov/geo/query/acc.cgi?acc=GSE41613).

## References

[1] Wang S, Yin S, Zhang ZL, Su X, Xu ZF (2019). Quality of life after oral cancer resection and free flap reconstruction. J. Oral Maxillofac. Surg.: Off. J. Am. Assoc. Oral Maxillofac. Surg..

[2] Gellrich NC, Handschel J, Holtmann H, Krüskemper G (2015). Oral cancer malnutrition impacts weight and quality of life. Nutrients.

[3] Fitzmaurice C (2019). Global, regional, and national cancer incidence, mortality, years of life lost, years lived with disability, and disability-adjusted life-years for 29 cancer groups, 1990–017: A systematic analysis for the global burden of disease study. JAMA Oncol..

[4] Ren ZH, Hu CY, He HR, Li YJ, Lyu J (2020). Global and regional burdens of oral cancer from 1990 to 2017: Results from the global burden of disease study. Cancer Commun. (Lond., Engl.).

[5] Zanoni DK (2019). Survival outcomes after treatment of cancer of the oral cavity (1985–2015). Oral Oncol..

[6] Luryi AL (2015). Treatment factors associated with survival in early-stage oral cavity cancer: Analysis of 6830 cases from the national cancer data base. JAMA Otolaryngol—Head Neck Surg..

[7] Moeckelmann N (2018). Prognostic implications of the 8th edition American joint committee on cancer (AJCC) staging system in oral cavity squamous cell carcinoma. Oral Oncol..

[8] Widschwendter M (2018). Epigenome-based cancer risk prediction: Rationale, opportunities and challenges. Nat. Rev. Clin. Oncol..

[9] Teschendorff AE, Relton CL (2018). Statistical and integrative system-level analysis of DNA methylation data. Nat. Rev. Genet..

[10] Portela A, Esteller M (2010). Epigenetic modifications and human disease. Nat. Biotechnol..

[11] Heyn H, Esteller M (2012). DNA methylation profiling in the clinic: Applications and challenges. Nat. Rev. Genet..

[12] Hinoue T (2012). Genome-scale analysis of aberrant DNA methylation in colorectal cancer. Genome Res..

[13] Villanueva A (2015). DNA methylation-based prognosis and epidrivers in hepatocellular carcinoma. Hepatology (Baltimore, Md.).

[14] Jiang H (2020). DNA methylation markers in the diagnosis and prognosis of common leukemias. Signal Transduct. Target. Ther..

[15] Klughammer J (2018). The DNA methylation landscape of glioblastoma disease progression shows extensive heterogeneity in time and space. Nat. Med..

[16] Hanahan D, Coussens LM (2012). Accessories to the crime: Functions of cells recruited to the tumor microenvironment. Cancer Cell.

[17] Vitale I, Manic G, Coussens LM, Kroemer G, Galluzzi L (2019). Macrophages and metabolism in the tumor microenvironment. Cell Metab..

[18] Anderson KG, Stromnes IM, Greenberg PD (2017). Obstacles posed by the tumor microenvironment to T cell activity: A case for synergistic therapies. Cancer Cell.

[19] Langevin SM (2012). Peripheral blood DNA methylation profiles are indicative of head and neck squamous cell carcinoma: An epigenome-wide association study. Epigenetics.

[20] Binnewies M (2018). Understanding the tumor immune microenvironment (TIME) for effective therapy. Nat. Med..

[21] Basu B (2017). Genome-wide DNA methylation profile identified a unique set of differentially methylated immune genes in oral squamous cell carcinoma patients in India. Clin. Epigenetics.

[22] Das D (2019). Epigenomic dysregulation-mediated alterations of key biological pathways and tumor immune evasion are hallmarks of gingivo-buccal oral cancer. Clin. Epigenetics.

[23] Zhu Q, Tian G, Gao J (2019). Construction of prognostic risk prediction model of oral squamous cell carcinoma based on co-methylated genes. Int. J. Mol. Med..

[24] Chang WC (2019). A histopathological evaluation and potential prognostic implications of oral squamous cell carcinoma with adverse features. Oral Oncol..

[25] Cao R, Yuan L, Ma B, Wang G, Tian Y (2020). Immune-related long non-coding RNA signature identified prognosis and immunotherapeutic efficiency in bladder cancer (BLCA). Cancer Cell Int..

[26] Kanehisa M, Goto S (2000). KEGG: Kyoto encyclopedia of genes and genomes. Nucleic Acids Res..

[27] Wang T (2017). T-cell receptor signaling activates an ITK/NF-κB/GATA-3 axis in T-cell lymphomas facilitating resistance to chemotherapy. Clin. Cancer Res.: Off. J. Am. Assoc. Cancer Res..

[28] Fesnak AD, June CH, Levine BL (2016). Engineered T cells: The promise and challenges of cancer immunotherapy. Nat. Rev. Cancer.

[29] Ribas A, Wolchok JD (2018). Cancer immunotherapy using checkpoint blockade. Science (New York, N.Y.).

[30] Andrews LP, Marciscano AE, Drake CG, Vignali DA (2017). LAG3 (CD223) as a cancer immunotherapy target. Immunol. Rev..

[31] Daley D (2017). Dectin 1 activation on macrophages by galectin 9 promotes pancreatic carcinoma and peritumoral immune tolerance. Nat. Med..

[32] Das M, Zhu C, Kuchroo VK (2017). Tim-3 and its role in regulating anti-tumor immunity. Immunol. Rev..

[33] Chauvin JM, Zarour HM (2020). TIGIT in cancer immunotherapy. J. Immunother. Cancer.

[34] You JS, Jones PA (2012). Cancer genetics and epigenetics: Two sides of the same coin?. Cancer Cell.

[35] Worsham MJ (2014). Delineating an epigenetic continuum in head and neck cancer. Cancer Lett..

[36] Milutin Gašperov N (2020). DNA methylome distinguishes head and neck cancer from potentially malignant oral lesions and healthy oral mucosa. Int. J. Mol. Sci..

[37] Ghantous Y, Nashef A, Abu-Elnaaj I (2020). Epigenetic alterations associated with the overall survival and recurrence free survival among oral squamous cell carcinoma patients. J. Clin. Med..

[38] Sun R (2020). Evaluation of DNA methylation in matched oral swab and tissue specimens from Chinese patients with oral squamous cell carcinoma. Int. J. Oral Maxillofac. Surg..

[39] Cheng SJ (2017). Hypermethylated ZNF582 and PAX1 genes in oral scrapings collected from cancer-adjacent normal oral mucosal sites are associated with aggressive progression and poor prognosis of oral cancer. Oral Oncol..

[40] Cheng SJ (2018). Hypermethylated ZNF582 and PAX1 genes in mouth rinse samples as biomarkers for oral dysplasia and oral cancer detection. Head Neck.

[41] Hoadley KA (2018). Cell-of-origin patterns dominate the molecular classification of 10,000 tumors from 33 types of cancer. Cell.

[42] Gao H (2020). Genome-wide DNA methylome analysis reveals methylation subtypes with different clinical outcomes for acute myeloid leukemia patients. Cancer Med..

[43] Yang X, Gao L, Zhang S (2017). Comparative pan-cancer DNA methylation analysis reveals cancer common and specific patterns. Brief. Bioinform..

[44] Toyota M (1999). CpG island methylator phenotype in colorectal cancer. Proc. Natl. Acad. Sci. U.S.A..

[45] Hughes LA (2013). The CpG island methylator phenotype: what’s in a name?. Can. Res..

[46] Hoivik EA (2013). DNA methylation of alternative promoters directs tissue specific expression of Epac2 isoforms. PLoS ONE.

[47] Ando M (2019). Chromatin dysregulation and DNA methylation at transcription start sites associated with transcriptional repression in cancers. Nat. Commun..

[48] Illingworth RS (2010). Orphan CpG islands identify numerous conserved promoters in the mammalian genome. PLoS Genet..

[49] Bae MG, Kim JY, Choi JK (2016). Frequent hypermethylation of orphan CpG islands with enhancer activity in cancer. BMC Med. Genom..

[50] Illingworth R (2008). A novel CpG island set identifies tissue-specific methylation at developmental gene loci. PLoS Biol..

[51] Carninci P (2006). Genome-wide analysis of mammalian promoter architecture and evolution. Nat. Genet..

[52] Demircioğlu D (2019). A pan-cancer transcriptome analysis reveals pervasive regulation through alternative promoters. Cell.

[53] Long J (2019). DNA methylation-driven genes for constructing diagnostic, prognostic, and recurrence models for hepatocellular carcinoma. Theranostics.

[54] Larmonie NSD (2018). MN1 overexpression is driven by loss of DNMT3B methylation activity in inv(16) pediatric AML. Oncogene.

[55] Shen Z (2019). ESRRG promoter hypermethylation as a diagnostic and prognostic biomarker in laryngeal squamous cell carcinoma. J. Clin. Lab. Anal..

[56] Virani S (2015). NDN and CD1A are novel prognostic methylation markers in patients with head and neck squamous carcinomas. BMC Cancer.

[57] Weiss D, Basel T, Sachse F, Braeuninger A, Rudack C (2011). Promoter methylation of cyclin A1 is associated with human papillomavirus 16 induced head and neck squamous cell carcinoma independently of p53 mutation. Mol. Carcinog..

[58] Xing X (2013). The prognostic value of CDKN2A hypermethylation in colorectal cancer: A meta-analysis. Br. J. Cancer.

[59] Kel A (2019). Walking pathways with positive feedback loops reveal DNA methylation biomarkers of colorectal cancer. BMC Bioinform..

[60] Heo WI (2017). Identification of novel candidate variants including COL6A6 polymorphisms in early-onset atopic dermatitis using whole-exome sequencing. BMC Med. Genet..

[61] Meyers KJ (2007). Genetic variations associated with echocardiographic left ventricular traits in hypertensive blacks. Hypertension (Dallas, Tex.: 1979).

[62] Pacifico R, Davis RL (2017). Transcriptome sequencing implicates dorsal striatum-specific gene network, immune response and energy metabolism pathways in bipolar disorder. Mol. Psychiatr..

[63] Gari MA (2016). Identification of novel genetic variations affecting osteoarthritis patients. BMC Med. Genet..

[64] VinuÉ Á (2019). Changes in CDKN2A/2B expression associate with T-cell phenotype modulation in atherosclerosis and type 2 diabetes mellitus. Trans. Res.: J. Lab. Clin. Med..

[65] Klajic J (2014). DNA methylation status of key cell-cycle regulators such as CDKNA2/p16 and CCNA1 correlates with treatment response to doxorubicin and 5-fluorouracil in locally advanced breast tumors. Clin. Cancer Res.: Off. J. Am. Assoc. Cancer Res..

[66] Jithesh PV (2013). The epigenetic landscape of oral squamous cell carcinoma. Br. J. Cancer.

[67] Sailer V (2019). DNA methylation of indoleamine 2,3-dioxygenase 1 (IDO1) in head and neck squamous cell carcinomas correlates with IDO1 expression, HPV status, patients’ survival, immune cell infiltrates, mutational load, and interferon γ signature. EBioMedicine.

[68] Arantes LM (2015). Validation of methylation markers for diagnosis of oral cavity cancer. Eur. J. Cancer (Oxford, England: 1990).

[69] Price WN, Cohen IG (2019). Privacy in the age of medical big data. Nat. Med..

[70] Miao YR (2020). ImmuCellAI: A unique method for comprehensive T-cell subsets abundance prediction and its application in cancer immunotherapy. Adv. Sci. (Weinh., Baden-Wurtt., Ger.).

