# Supplementary material for: Prognostic and therapeutic prediction by screening signature combinations from transcriptome–methylome interactions in oral squamous cell carcinoma
Source: Sci Rep. 2022 Jul 6;12:11400. doi: 10.1038/s41598-022-15534-7 (PMC9259703; doi:10.1038/s41598-022-15534-7)

**Prognostic and therapeutic prediction by screening signature combinations from transcriptome–methylome interactions in oral squamous cell carcinoma**

**Supplemental materials**

**Table S1**. Summary of clinicopathological information of analyzed individuals.

**Figure S1**. Scatter plots of CpGs methylation grouped by related gene expression quartiles in either tumor or normal samples (n = 5493, 5493, 5493, 5496 for Q1, Q2, Q3, Q4, respectively; Q4 is the highest expression) showing methylation ratio at 100 bp segments within and outside CGI.

**Figure S2.** Plots of the median methylation values per 100 bp distance grouped by gene expression quartiles based on the expression levels in paired normal samples, paired Tumor samples, and single normal samples.

**Figure S3**. Heatmap of correlations among immune cell infiltrations.

**Figure S4**. Box plots of immune-checkpoint-relevant gene expressions of PDCD1LG2 and CD274.

**Figure S5**. Differential chemotherapeutic responses in high- and low-risk patients to chemotherapeutic drugs.

**Table S1**. Summary of clinicopathological information of analyzed individuals.

|  | Mean (range) or number (ratio) | N |
| --- | --- | --- |
| Age | 60.0 (52.0 - 69.0) | 390 |
| Gender |  | 391 |
| Female | 119(30.4%) |  |
| Male | 272(69.6%) |  |
| Alcohol |  | 391 |
| No | 121(30.9%) |  |
| Not reported | 9(2.30%) |  |
| Yes | 261(66.8%) |  |
| Frequency of smoking |  | 381 |
| 1 | 112(29.4%) |  |
| 2 | 115(30.2%) |  |
| 3 | 58(15.2%) |  |
| 4 | 94(24.7%) |  |
| 5 | 2(0.52%) |  |
| pT |  | 347 |
| T1 | 40(11.5%) |  |
| T2 | 122(35.2%) |  |
| T3 | 72(20.7%) |  |
| T4 | 113(32.6%) |  |
| pN |  | 317 |
| N0 | 133(42.0%) |  |
| N1 | 55(17.4%) |  |
| N2 | 124(39.1%) |  |
| N3 | 5(1.58%) |  |
| pM |  |  |
| M0 | 139(100%) | 139 |
| cStage |  | 381 |
| Stage I | 17(4.46%) |  |
| Stage II | 86(22.6%) |  |
| Stage III | 77(20.2%) |  |
| Stage IV | 201(52.8%) |  |
| Grade |  | 374 |
| G1 | 54(14.4%) |  |
| G2 | 228(61.0%) |  |
| G3 | 86(23.0%) |  |
| G4 | 6(1.60%) |  |
| HPV |  | 95 |
| Negative | 57(60.0%) |  |
| Positive | 38(40.0%) |  |
| Neck dissection |  | 389 |
| NO | 70(18.0%) |  |
| YES | 319(82.0%) |  |
| Lymphovascular invasion |  | 266 |
| NO | 180(67.7%) |  |
| YES | 86(32.3%) |  |
| Margin |  | 354 |
| Close | 43(12.1%) |  |
| Negative | 262(74.0%) |  |
| Positive | 49(13.8%) |  |
| Perineural invasion |  | 283 |
| NO | 141(49.8%) |  |
| YES | 142(50.2%) |  |


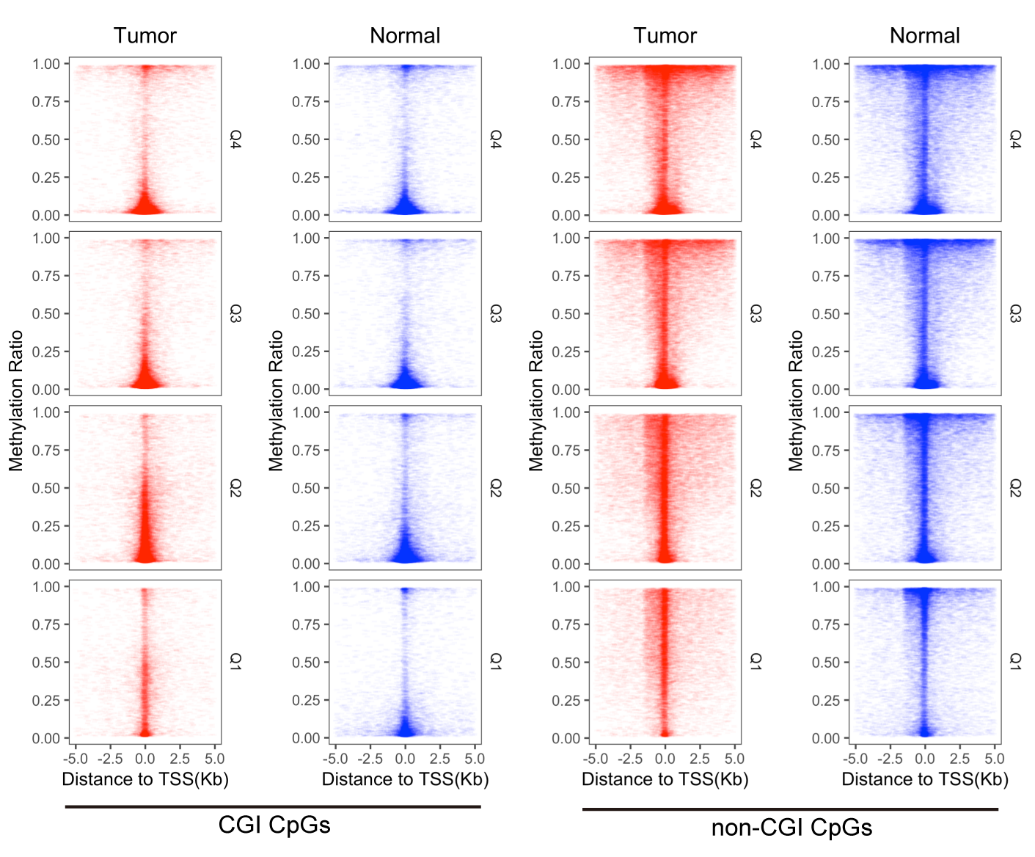
**Figure S1**. Scatter plots of CpGs methylation grouped by related gene expression quartiles in either tumor or normal samples (n = 5493, 5493, 5493, 5496 for Q1, Q2, Q3, Q4, respectively; Q4 is the highest expression) showing methylation ratio at 100 bp segments within and outside CGI

**Figure S2.** Plots of the median methylation values per 100 bp distance grouped by gene expression quartiles based on the expression levels in paired normal samples, paired Tumor samples, and single normal samples.


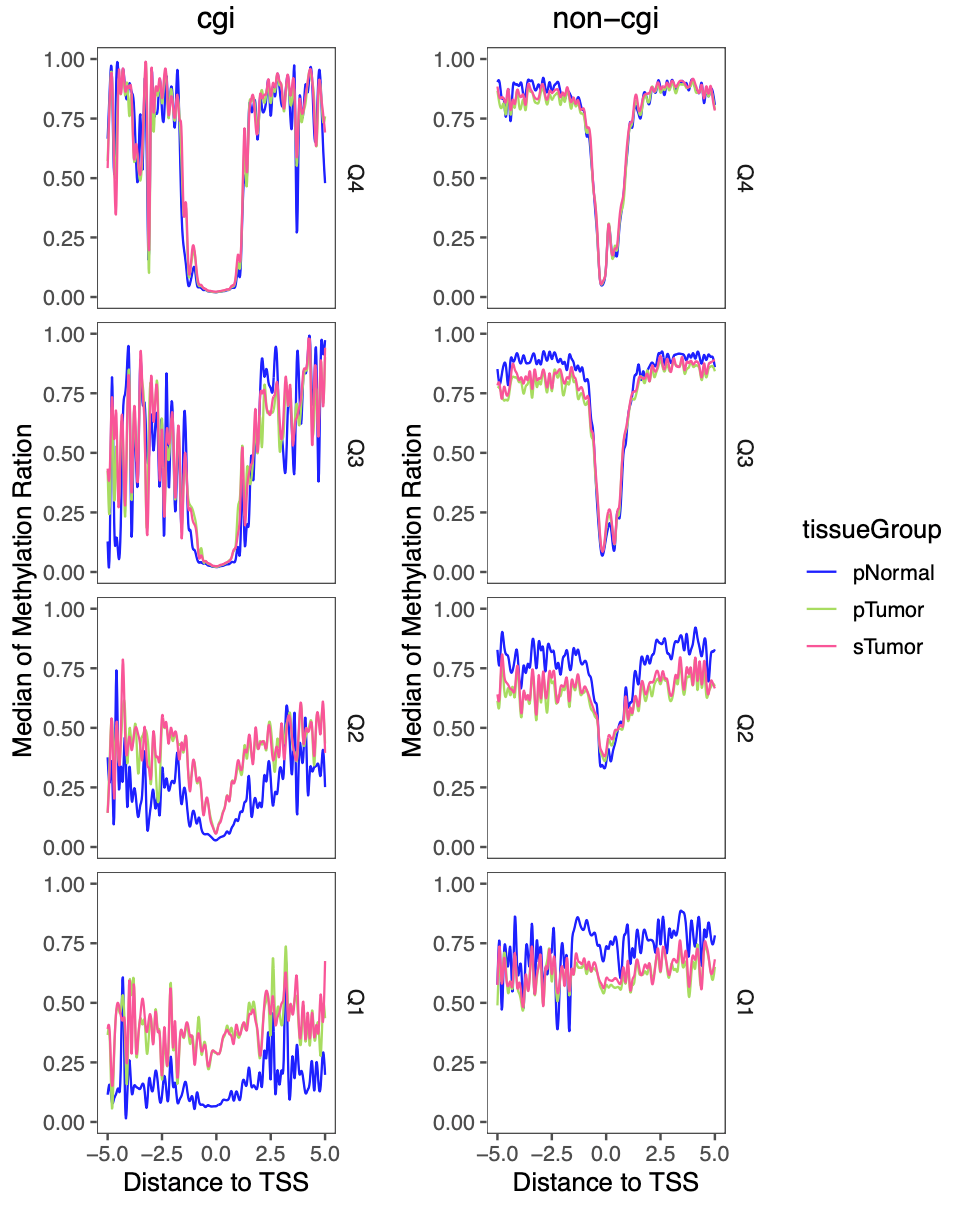


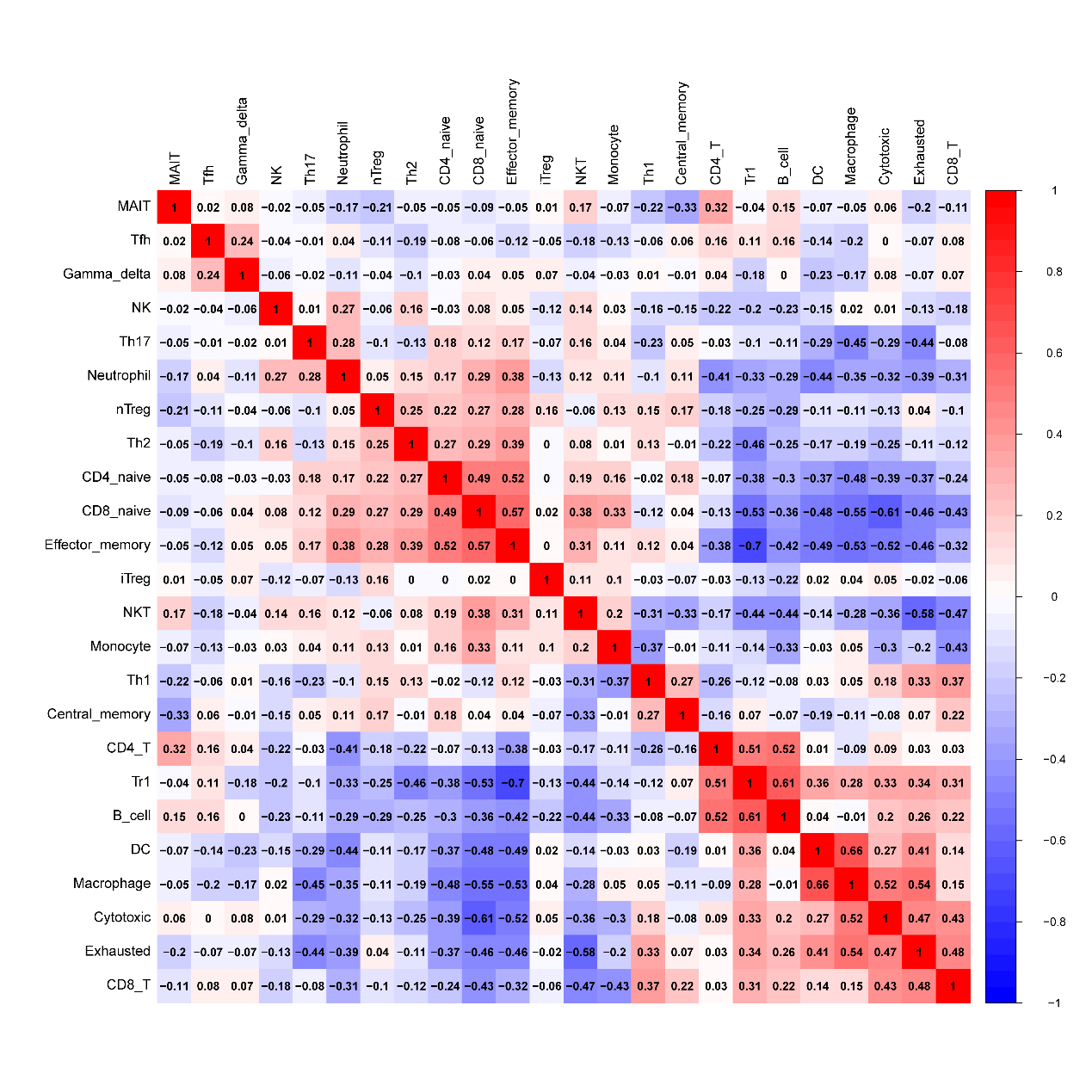
**Figure S3**. Heatmap of correlations among immune cell infiltrations.

**Figure S4**. Box plots of immune-checkpoint-relevant gene expressions of PDCD1LG2 and CD274.


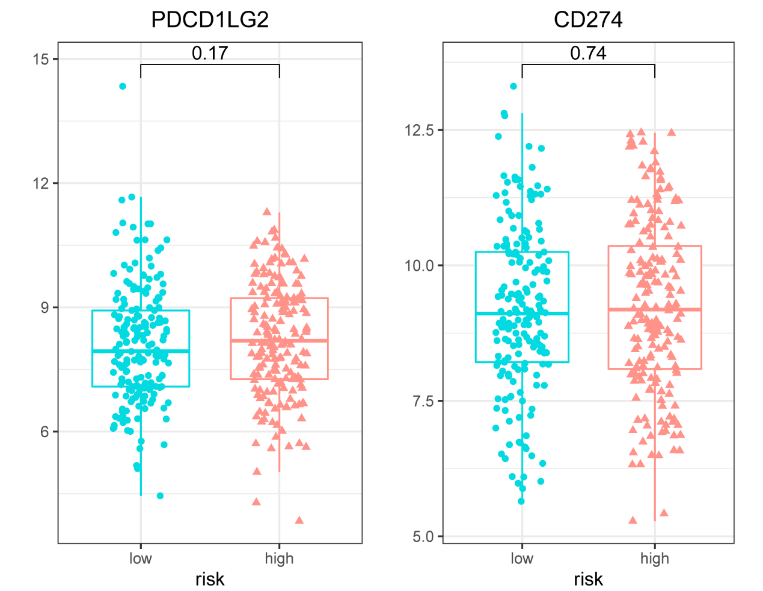


**Figure S5**. Differential chemotherapeutic responses in high- and low-risk patients to chemotherapeutic drugs.


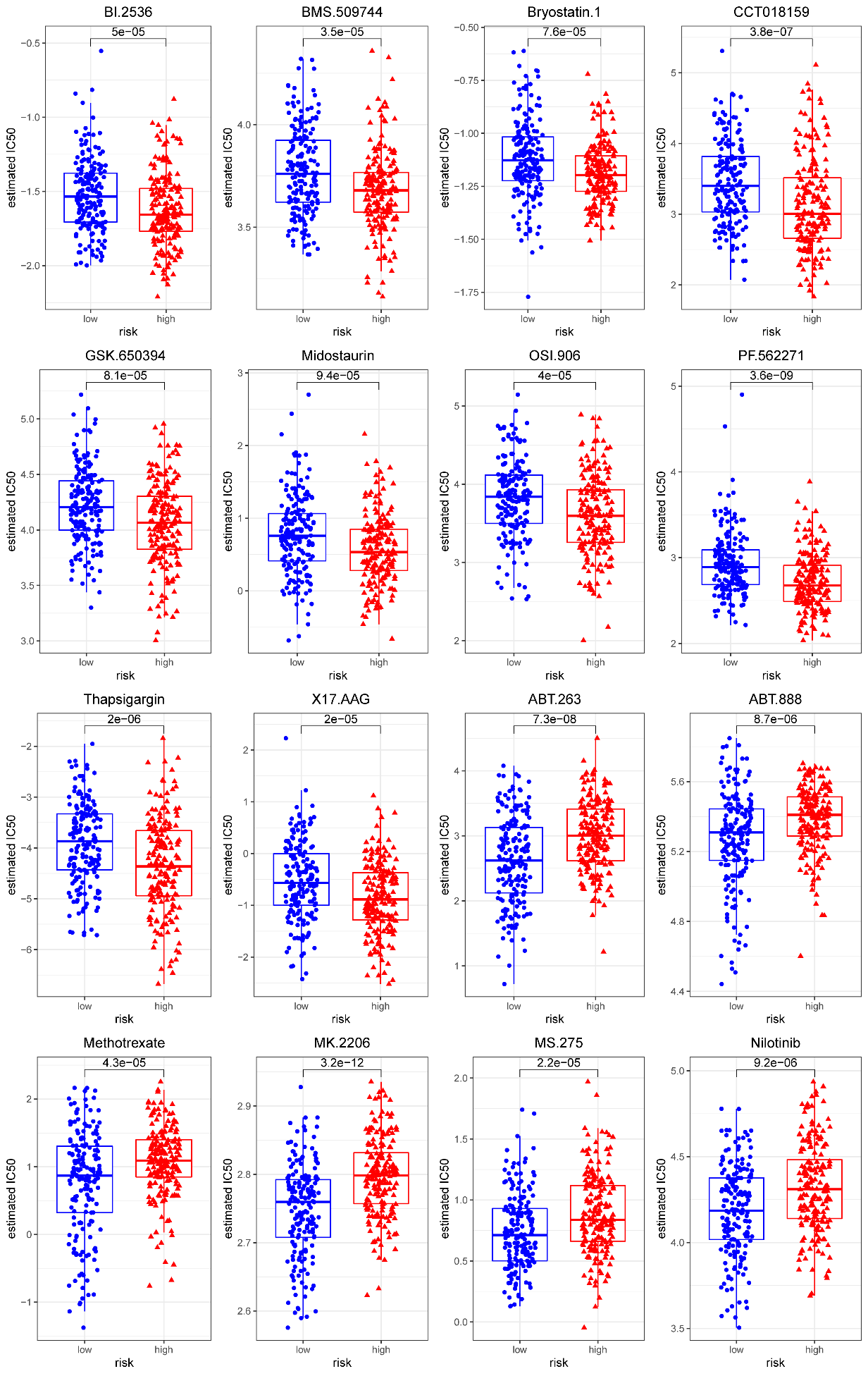

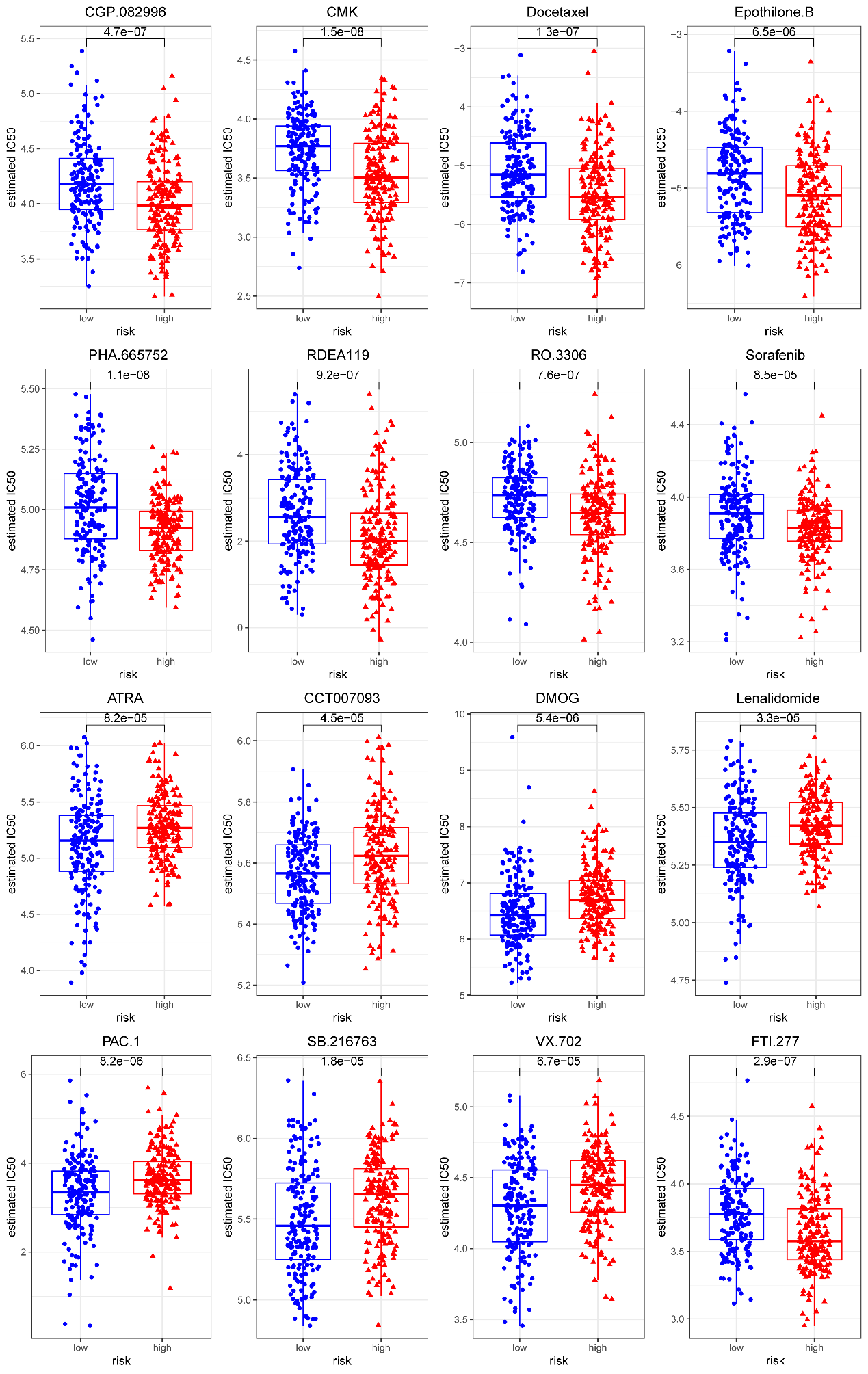

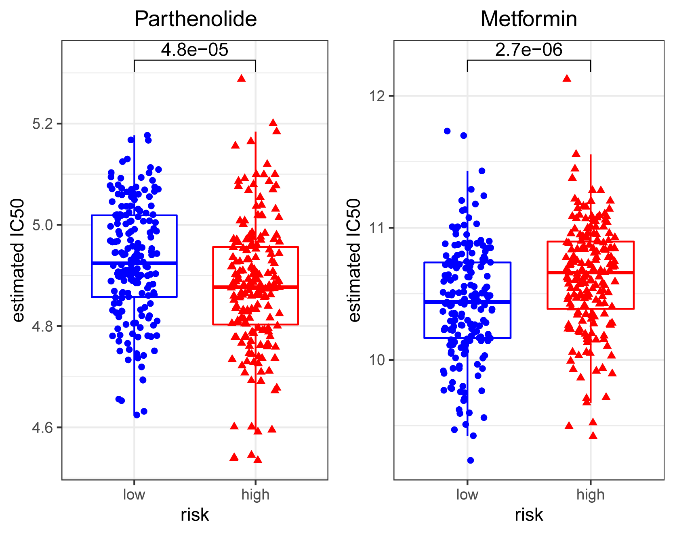

Supplement: Supplementary file 1 — Supplementary Information. [file 41598_2022_15534_MOESM1_ESM.docx]
